# The Road Not Taken with Pyrrole-Imidazole Polyamides: Off-Target Effects and Genomic Binding

**DOI:** 10.3390/biom10040544

**Published:** 2020-04-03

**Authors:** Jason Lin, Hiroki Nagase

**Affiliations:** Laboratory of Cancer Genetics, Chiba Cancer Center Research Institute, Chiba 260-8717, Japan

**Keywords:** *N*-heterocycles, minor-groove binding, pyrrole-imidazole polyamides, genomics, chemical biology

## Abstract

The high sequence specificity of minor groove-binding *N*-methylpyrrole-*N*-methylimidazole polyamides have made significant advances in cancer and disease biology, yet there have been few comprehensive reports on their off-target effects, most likely as a consequence of the lack of available tools in evaluating genomic binding, an essential aspect that has gone seriously underexplored. Compared to other *N*-heterocycles, the off-target effects of these polyamides and their specificity for the DNA minor groove and primary base pair recognition require the development of new analytical methods, which are missing in the field today. This review aims to highlight the current progress in deciphering the off-target effects of these *N*-heterocyclic molecules and suggests new ways that next-generating sequencing can be used in addressing off-target effects.

## 1. Introduction

**Figure 1:**
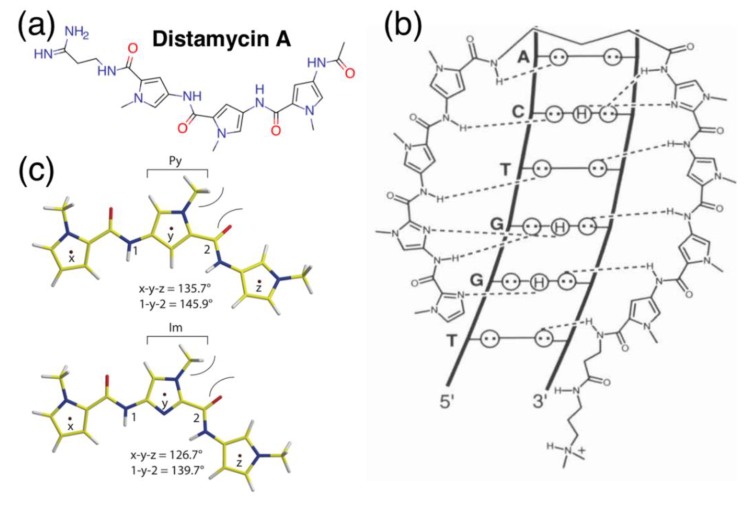

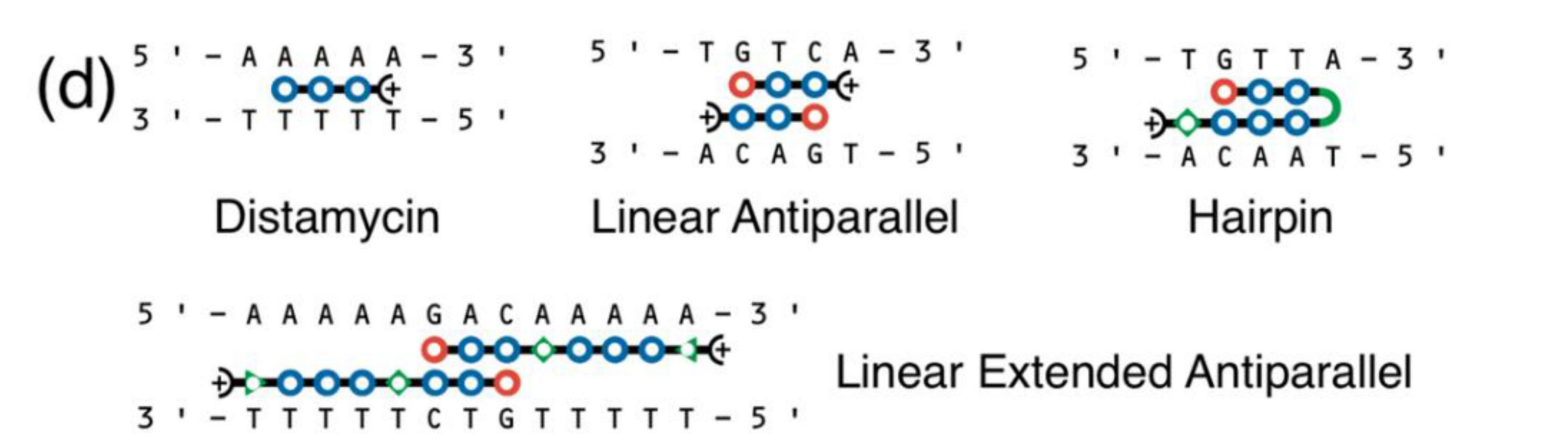
Schematics of pyrrole-imidazole polyamides and DNA binding. (**a**) Structure of distamycin A. (**b**) Illustrative model of hydrogen-bonding interactions between pyrrole-imidazole polyamides (PIPs) and the DNA minor groove; circles with dots represent lone pairs of purines and pyrimidines; circles containing an H represent the additional hydrogen on G; putative hydrogen bonds are indicated with dotted lines. Reprinted with permission from [1], © 2001 American Chemical Society. (**c**) Local conformation modeling of *N*-methylpyrroles (Py, top) and *N*-methylimidazoles (Im, bottom) in PIP backbones. Bond angles x-y-z and 1-y-2 are indicated directly below. Reprinted with permission from [15], © 2011 American Chemical Society. (**d**) Example of various binding models’ PIP-DNA minor-groove complexes: linear antiparallel, hairpin and linear extended antiparallel. Red and blue circles represent imidazole and pyrrole rings, respectively; green triangles, diamonds, curved green lines and +) represent glycine, β-alanine, ɣ-aminobutyric acid and the *N*,*N*-dimethylaminopropylamide (Dp) capping, respectively. Reprinted from [16], © 1996, with permission from Elsevier.

*N*-methylpyrrole-*N*-methylimidazole polyamides (pyrrole-imidazole polyamides, PIPs) possess the incredible ability to interact and bind the DNA minor groove with programmable specificities exceeding distamycin (Figure 1a), the molecule that sparks the inspiration behind this field. These heterocycles bind and are able to differentiate their respective nucleosides to single hydrogen-bond precisions (Figure 1b, [1]) and are typically generated by solid-phase [2] or, at times, solution-phase synthesis [3]; the production of PIPs can be readily scaled up to milligram scales for a variety of different biological applications. Recent studies on PIP have been investing on the possible applications in silencing “undruggable” biological targets that are difficult to inhibit at the protein level. Those so-called undruggable targets present challenges of drug sensitivity and resistance because of their intracellular localization and numerous associations with other proteins and co-factors in macromolecular complexes [4]. In contrast, upon minor-groove binding, these polyamides will effectively silence their respective marks by disrupting transcription, bypassing issues associated with the usual approach of inhibition via the binding pocket or surface access. This unique biochemical feature makes PIPs especially attractive as a medium of mitigating the virulence of oncological targets, a number of which have arisen as a consequence of tens to millions of genetic and chromosomal aberrations harbored within the cancer genome [5,6].

Currently, a significant portion of research in PIPs is centralized on lead developments; this relocates the focus squarely onto the exploration of possible targets in diseases and biological systems. There is significant focus on the chemistry and the effects of chemical modifications on these polyketides in terms of improving their pharmacokinetic parameters, such as the tuning of lipophilicity and motif recognition. While most of these results have been promising, an area severely underinvestigated is the possibility of off-target bindings and their potential implications. These off-target-binding events lead to the rise of adverse effects as other genes are transcriptionally impacted, subsequently triggering changes to other biological processes which may be totally unrelated to the target of interest [7]. It is but a certain mathematical possibility that all PIPs currently in development will bind multiple targets as a consequence of their mode of motif recognition. Suppose the probability that a particular *k*-mer occupies a particular genomic location is (*4k*)^−1^; accounting for DNA being double-stranded, we can reasonably posit that the expected number of *k*-mer repeats in the human genome is approximately *2N^2^/4^k^*, with *N* being roughly three billion. For the expectation to fall close to 0, a back-of-the-envelope calculation would reveal that, at a minimum, we need to achieve a base recognition rate beyond 32 to 36 bases. With siRNAs, which tend to be 20–25-bases-long, the expectation that there is only one binding site is a mathematical impossibility; PIPs and their usual span of recognition being 5 to 10 nucleotides are also out of the range of uniqueness. While efforts to lengthen PIP recognition by tandem conjugation have succeeded [8,9], with the molecular size, it very quickly reaches near the ceiling of the nuclear diffusion limit of ~10 nm for macromolecules [10,11].

This “unfortunate” aspect of being unable to reach uniqueness, however, does not discount the usefulness of PIPs. Even with CRISPR/Cas9 systems, there is nonetheless a need to extend guide RNAs beyond the same limit of uniqueness, not to mention the myriad of undesired effects such as exon-skipping and truncated expressions [12] or recent controversies on using such a genome-editing tool on human subjects. These concerns squarely place PIPs back into the focus as therapeutic alternatives for various diseases, but along the same vein, one cannot simply disregard the likelihood of off-target binding and the resulting consequence of these binding events. Unlike other nitrogen heterocycles that find their way onto the list of 59% of approved drugs in the United States [13] or DNA-binding drugs such as cisplatin, doxorubicin or cryptolepine [14], PIPs inherently “bundle” programmability, with the benefit of having fewer off-target sites by the virtue of their lengthened motif recognition; yet, those other molecules see extensive research in their adverse indications, while the field for PIPs is paradoxically barren. Why are there so few willing to venture down this path? This review aims to discuss the feasibility of PIPs as pharmaceutical leads, outline some of the current challenges in the evaluation of off-target binding in these molecules and comment on the possible future directions in exploring off-target binding.

## 2. Biological Applications of PIPs

**Figure 2:**
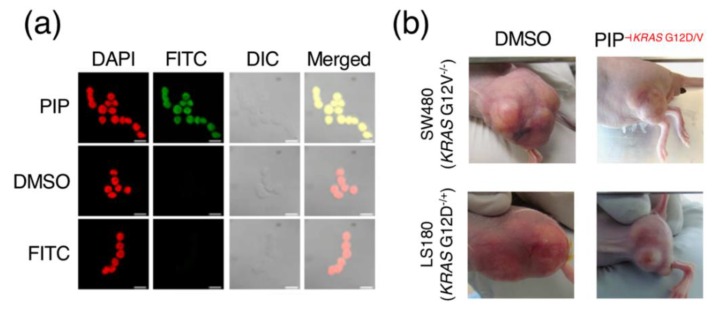
Biological applications of PIPs. (**a**) Example of in-situ cell imaging using a fluorescein isothiocyanate (FITC)-conjugated PIP; reproduced from [22], © 2018 with permission from Elsevier. (**b**) The coupling of 1-chloromethyl-5-hydroxy-1,2-dihydro-3H-benz(e)indole (CBI), an alkylating agent, to a PIP-targeting *KRAS* G12D/V mutation is able to reduce the size of tumors in human colorectal cancer LS180/SW480 xenograft mouse models; images shown here are representative of multiple specimens, with DMSO (left) as a control; reused from [25] by the author, © 2015 Springer Nature Limited.

Pyrrole-imidazole polyamides (PIPs) themselves occupy a corner of naturally inspired antibiotic mimetics; these *N*-heterocycles consist of a backbone of *N*-methylpyrrole and *N*-methylimidazole subunits (Figure 1c) assembled together by amide bonds [15] and are able to interact with the DNA minor groove in various structural conformations (Figure 1d), with the hairpin [16] configuration being one of the most commonly adopted. Despite the simplicity behind its chemistry, the amide bonds in PIP backbones permit the conjugation of a variety of different reactive moieties that diversify the structure and functionalities of PIPs. Their innate ability to bind the DNA minor groove allows them to be used to repress gene transcription via triggering RNA polymerase II inhibition [17], without having to resort to unstable or complex experimental procedures such RNAi or CRISPR/Cas. As such, by binding the minor groove, one can avoid the messiness of conventional small molecular inhibitors that work on protein surfaces. PIPs have been used in a variety of biological applications; in 2005, Beerman and colleagues successfully demonstrated the ability to cause DNA damage by conjugating 1-chloromethyl-5-hydroxy-1,2-dihydro-3H-benz(e)indole (CBI), an alkylating agent, to disrupt and inhibit SV40 DNA replication [18]; conjugation of other functional groups have also led to the rise of additional functionalities for PIPs, such as artificial nucleases [19], gene switches [20], transcriptional regulators via histone acetyltransferase activators [21] and imaging probes [22] upon the conjugation of fluorescent moieties (Figure 2a) or radioisotopes [23]. Even without those modifications, PIPs are still able to retain their high specificity, unlike mainstream DNA-damaging agents such as cisplatin [17].

The relative ease and robustness of synthesis, as well as the capability to conjugate functional groups by the same amide chemistry, is perhaps the most important feature that has brought PIPs to the forefront of chemical biology. The ability to target select regions of the genome and exert specific actions such as transcriptional disruption, epigenetic reprogramming or the recruitment of transcriptional elements has led to the development of a number of unique and interesting biological applications for PIPs, especially in the areas of cancer and disease biology. This also theoretically simplifies and increases the throughput of the design process. PIPs have their ways in the inhibition of oncological targets such as MMP [24], constitutively active *KRAS* [25], a frequent driver gene in cancers such as colorectal, lung and pancreatic cancers, as well as members of the *RUNX* transcription factor family [26]. Notably, the PIP-targeting G12D/V mutation in *KRAS* effectively restrained tumor growth in a mouse xenograft model (Figure 2b). PIPs have also shown effectiveness in targeting genetic aberrations other than single-base mutations, such as the case with copy-amplified *MYCN* [27], an aberration in neuroblastoma, where appreciable DNA damage and induced apoptosis were observable in *MYCN*-amplified neuroblastoma mouse models. There have also been reports of PIPs suppressing transcription factors central to hypoxic gene expressions relevant in neovascularization and cancer metastasis [28], as well as demonstrating antiproliferative activity in prostate cancer cells via the modulation of the androgen receptor [29,30] in ways different from other drugs such as camptothecin, doxorubicin or etoposide [31]. Some of the other examples include the ability to interfere with TNF-α-inducible transcriptome [32] and the triggering of inflammatory necrotic cell death in cancer cells via calreticulin, ATP and HMGB1 signaling [33]. Beyond cell-level and mouse model experiments, preclinical trials in marmosets also show promising success of a PIP targeting the human TGF-β1 promoter in reducing scarring [34].

## 3. Off-Target Effects and “The Road Not Taken”

Despite the stellar performance of PIPs in biological applications, this class of molecules has yet to see commercial success as leads in drug discovery. In theory, one can systematically design, synthesize and test a larger number of PIPs with relative ease, possibly even over compounds derived from combinatorial chemistry. With their specificity and unique ability to penetrate nucleus and target DNA compared to conventional broad-spectrum chemotherapy agents that are cytotoxic compounds with a large number of adverse indications, it is not unrealistic to expect the presence of multiple PIPs in clinical trials or, perhaps, even on the market today. There, however, seems to be little movement for PIPs in pharmaceutical chemistry. Furthermore, there have been few reports dedicated to the discussion of off-target effects, one of the most important factors in drug discovery; nearly 50% of the attritions during the United States Food and Drug Administration Phase II clinical trials are due to the occurrence of toxic side effects [35]. Understandably, most would prefer to steer conversations away from topics that could quickly turn to discount their own work, leading to tremendous undergrowth down this seldom-traversed route. Unfortunately, this remains a critical area of discussion, especially during lead discovery and optimization. Performing research in this area, however, is neither exactly pleasant nor encouraging. What, then, are off-target effects, and why do researchers not elect to investigate this path less traveled?

### 3.1. Defining and Evaluating “Off-Target” Effects

In the most general sense, binding to any genomic location other than a polyamide’s intended site can be considered off-target, and in the human genome, it is a mathematical certainty that PIPs will have off-target effects. This is not a problem strictly related to PIPs, however; even revolutionary genome-editing systems like CRISPR/Cas are affected by this phenomenon; a recently discovered artifact in CRISPR/Cas, for even, is that genomic deletions induced by CRISPR may lead to unintended exon skipping and the production of aberrant proteins [12]. To attenuate these undesirable effects requires that we first understand *what* those effects are; unfortunately, our current knowledge in the subject matter is practically nonexistent. If we could understand these off targets, we could then design new PIP candidates that try not to target these genomic regions or make chemical modifications that alter the affinity of PIP-DNA ligand interactions. Similarly, by understanding these off targets, we may be able to reposition PIPs for other applications in a manner not unlike others have done in pharmaceutical chemistry, such as Zidovudine [36] or Viagra^®^ [37].

**Figure 3:**
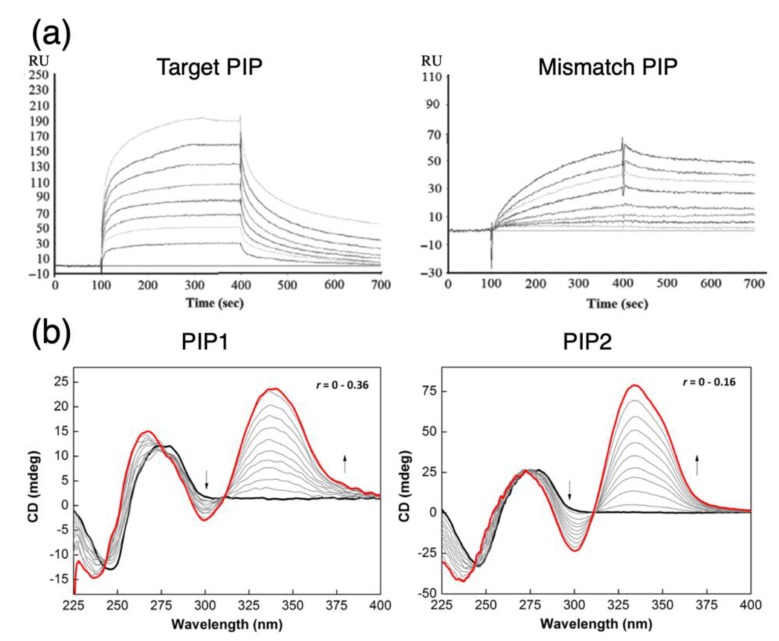

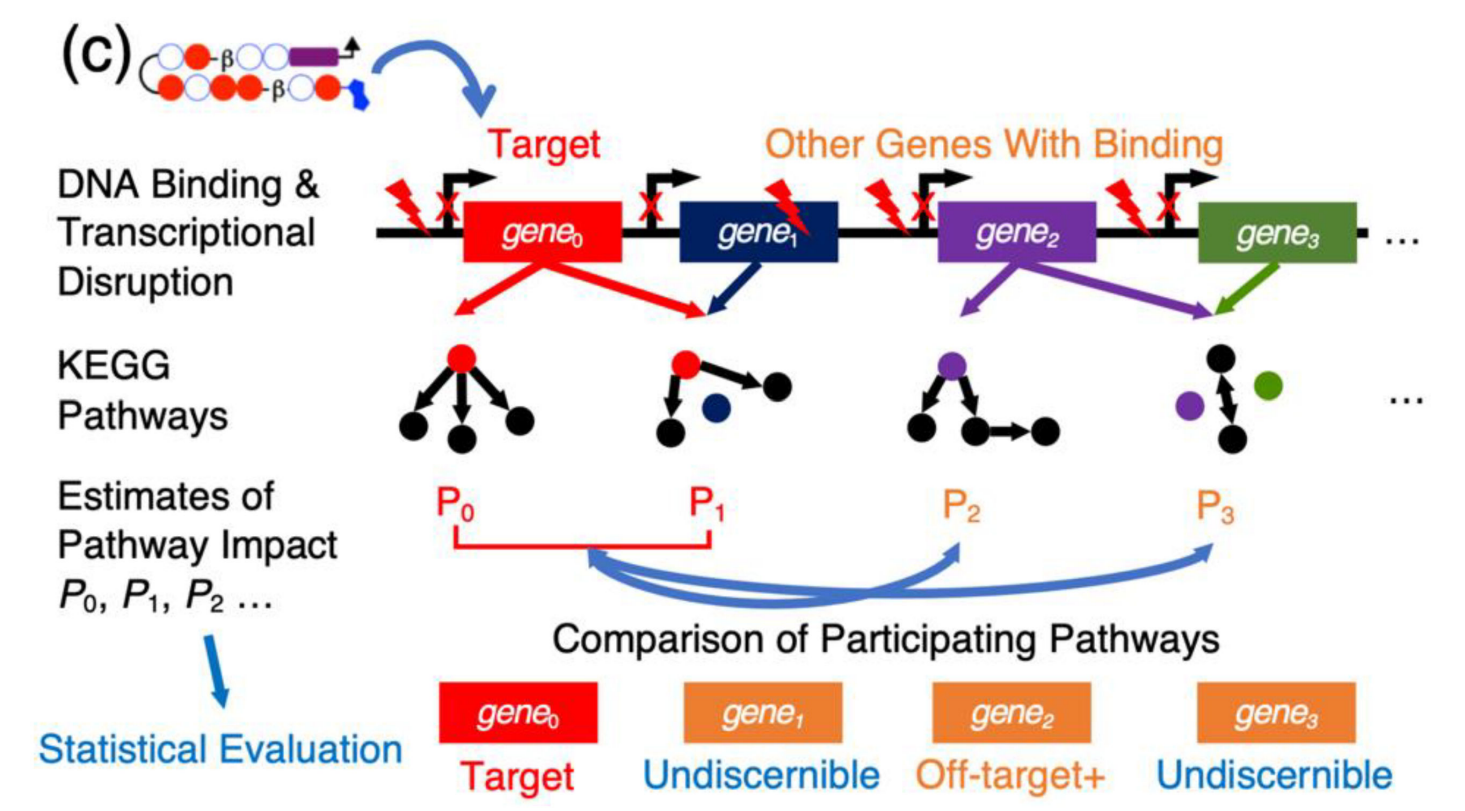
Analyzing off-target effects of PIPs with biophysical assays and expression profiling. (**a**) Illustrative use of surface plasmon resonance to compare relative binding specificities of PIPs to DNA containing a particular motif. Evaluation of the polyamides (“Target PIP” and “Mismatch PIP”) rely on differences in the association-dissociation kinetics; equilibrium constants can be estimated based on the trajectories in the sensorgram to elucidate further details about how a particular design variation will affect binding and the extent of binding to mismatched sequences; reproduced from [48], © 2015 (license terms CC BY-NC-ND). (**b**) Illustrative use of circular dichroism to determine the relative difference in PIP-DNA interactions via the monitoring of local conformational changes in the minor groove. The presence of features such as isoelliptic points in the DNA-absorbing wavelength region (230–300 nm) and induced signals near 355 nm indicate a favorable interaction. Comparison of spectra of different PIP candidates allow comparative assessments to be made. Adapted with permission from [42], © 2015 American Chemical Society. (**c**) A proposed method of determining the candidate of off-target genes using expression profiling and the binding site (modified from [49], © 2019 (license terms CC BY); the method utilizes pathway information to reduce the dimensionality of expression arrays to estimate off-target effects at the pathway level (illustrated in the example as *P*_0_, …, *P*_3_). These metrics could then be used to compute overall changes that may be deconvoluted later on to identify potential off targets (“Off-target+” in orange) from genes that were somehow impacted as a consequence of other on-target and off-target genes in the pathway (“Undiscernible” in blue).

Currently, evaluating off-target effects primarily involves the use of various biochemical and biophysical assays (see examples in Table 1) to compare PIP binding with full-match and mismatch nucleotides. By understanding the differences in recognition affinity, we can deduce to some extent the likelihood of a PIP’s cross-reactivity with unintended regions of the genome, along with valuable biological insights on the mode of action of PIPs in vivo, such as the possible dependence on chromatin structure and histone modifications on DNA accessibility [38] to help characterize the properties of a candidate PIP. These assays typically provide us with some measures of quantitative differentiation, e.g., equilibrium association/dissociation rates (Figure 3a) via surface plasmon resonance (SPR) or preferential binding rates via digital polymerase chain reactions [39]. For instance, structural flexibilities near the termini of the polyamide backbone will substantially alter the association and dissociation rate constants to shift the binding affinity of the PIP towards mismatched DNA [40,41]. Qualitatively, gel-shift electrophoresis can also provide a preliminary and inexpensive assessment of the relative affinity of PIP-DNA ligands. Circular dichroism experiments (Figure 3b) can also elucidate additional biophysical insights, such as the influence on DNA binding as the minor groove becomes narrowed due to changes in ionic strength that ultimately restricts groove size and alters the electrostatic repulsion of the negatively charged phosphate groups [42]; in some cases, circular dichroism may also be used to determine whether particular functional groups have successfully been delivered or activated [43] to the proximity of target DNA.

Piecing together these molecular level clues about how PIPs behave, we may be able to derive a crude understanding of how these biochemical details will influence off-target binding; indeed, undoubtedly, changes in the local PIP-DNA ligand conformation will weaken this interaction and perchance destabilize nearby transcription elements, subsequently disrupting the transcription, although we may not be able to decipher what sort of phenotypic changes these can lead to. Nonetheless, one can safely posit that epigenetic changes are intimately linked to the rise of off-target effects. This is well-corroborated with the fact that small conformational distortions are sufficient to retard RNA polymerase II [17] and potentially displace histones [44]. Evidence also suggests that lipophilicity of a PIP is associated in tumor-directed delivery in situ [22], albeit there may be some dependence on the type and origin of tumors [45]. We can thus potentially expand our synthetic repertoire to introduce novel modifications to the existing PIP design toolbox to incorporate these improvements, but there are still caveats to take note of, not to mention again that these assays may only have provided us with qualitative results. For instance, further increasing a PIP’s lipophilicity may increase its propensity to aggregate, reducing its biocompatibility and restricting its ability for tissue penetration. Despite reports that the aggregation propensity of PIPs should not contribute to biological activity and may not be a critical concern in pharmacokinetic analyses [46] and conflicting reports suggesting possible liver toxicity with certain hairpin modifications [47] in mouse models at a dose of ~10 mg/kg, one needs to be aware that there are still limits to the amount of chemical modifications to apply to PIPs, and other resolutions need to be attempted to address this complex problem.

Aside from a PIP’s biophysical properties, arguably the most critical aspect in understanding what off-target effects are is the frequency and likelihood of genomic-binding events. Whereas, with protein-level inhibitors, one may be able to piece together structural information and interactions with other proteins to infer drug indications [50]. We can estimate a PIP’s binding sites by searching for its recognition motif in a sequence file, but to understand the frequency (and likelihood), more fine-grained approaches are required. A likely choice in this case is expression microarrays, for one can easily evaluate simultaneously changes in an entire genome with a single chip. However, changes in the expression profile may tell us what unexpected genes are up- or downregulated as a consequence of PIP administration, but they provide only a stochastic snapshot of the dynamic profile and cannot infer what actual mechanisms lead to the rise of these regulatory changes. Since chemical stimuli trigger environmental or oxidative stress homeostasis, those changes we observed may simply be a nonspecific response to having been perturbed by the mere event of PIP diffusion, complicating our understanding of the question we wish to address; how does one formulate a solution to such a question, then? A recent proposal is to reformulate the question to convolute the expression change of individual genes into units of pathways [49] as a mean of reducing the dimensions of analytical complexity (Figure 3c); as the problem is simplified, this clarifies the null hypothesis to evaluate and compare pathways that we expect to be modified compared to other pathways that are ordinarily unaffected. This analysis, however, still makes two underlying assumptions that remain to be verified: first, PIP-binding sites in regions that do not affect a gene’s expression probably have no effect; second, binding to those other sites is equally likely, such that all genes affect the outcomes equally. To evaluate whether those two assumptions are applicable, we will need the power of next-generation sequencing at our disposal.

### 3.2. Application of Next-Generation Sequencing in Evaluating PIP Off-Target Effects

Next-generation sequencing (NGS) methods provide a conduit to probe a PIP’s genomic interactions and, subsequently, address the question of off-target binding. When coupled with expression-profiling results, sequencing analysis of PIP binding can provide insights into the interaction of these minor groove-binding heterocycles in the genomic space via binding-site information and nearby epigenetic changes. There are currently two major directions of using NGS to address these questions: Bind-n-Seq, which primarily aims to resolve motif recognition, and Chem-seq, which utilizes affinity enrichment to capture information on genomic-binding sites.

#### 3.2.1. Bind-n-Seq: Inferences on Binding Motifs

**Figure 4:**
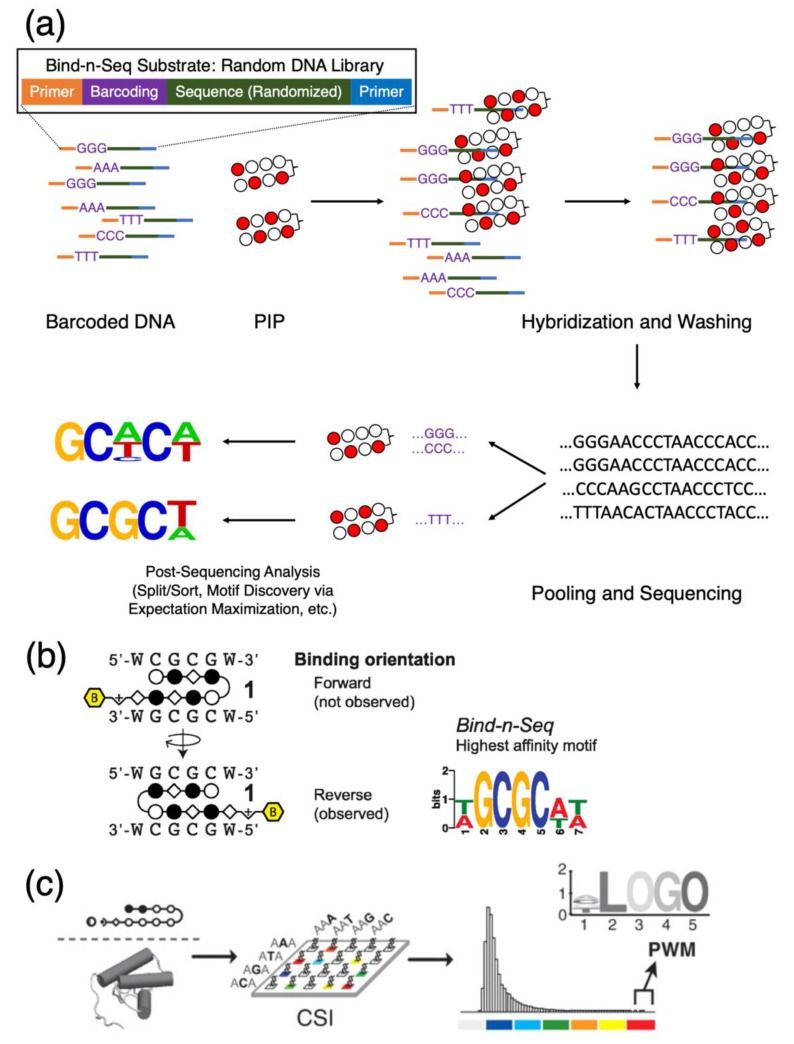
Mechanics of using Bind-n-Seq to elucidate the sequence specificity of PIPs. (**a**) Schematics of Bind-n-Seq, beginning with a random oligonucleotide library to create a possible sequence space for downstream analysis. (**b**) The ability to extract intricate details about PIP-binding modes with Bind-n-Seq; example shown here is the different in binding orientation. Reprinted with permission from [52], © 2014 American Chemical Society. (**c**) Cognate site identifier, or CSI, as a variation of a similar theme to identify binding motifs; motif enrichment results can then be transformed into position weight matrices (PWM) or presented as motif logo maps. Reproduced from [55], © 2010 National Academy of Sciences.

Bind-n-Seq is, as its name implies, is a sequencing method that involves the hybridization of a molecular target with DNA to form ligands and the subsequent sequencing of said ligands [51]. This method utilizes a library of barcoded synthetic and typically randomized oligonucleotides to interact with the molecular target in question. Upon affinity capture and hybridization, unbound nucleotides are removed by washing, pooled and sequenced (Figure 4a). Binding preferences in the form of motif logos or position-weight matrices are calculated based on the relative abundance of bound DNA. Bind-n-Seq studies are high in throughput and have become prevalent in PIP studies, as the resultant logos provide quantitative data between the relative affinity of a full-match motif compared to its mismatch counterparts. These sorts of experiments do not require a large number of reagents and allow simultaneous cross-comparisons of multiple biotinylated PIPs due to the adaptation of oligonucleotide barcoding; Dervan et al., for instance, were able to perform comparisons of eight PIPs with a single run of Bind-n-Seq [52]. This technique can also infer additional binding details out of the purview of biophysical assays; for example, the relative preference for binding orientations (for instance, see Figure 4b for the same study of polyamides targeting the 5′-CGCG-3′ sequence as the means of inhibiting CpG methylation). Bind-n-Seq is also compatible with PIPs functionalized with alkylating agents such as CBI that form covalent linkages with DNA via the adenine in the 3′ terminus of its recognition motif [53]; this makes the method a more preferable choice at times over SPR, as CBI-conjugated polyamides will react irreversibly with the immobilized oligonucleotide targets and prevent dissociation from the sensor chip.

Variations of Bind-n-Seq include Bind-n-Seq-MR, which incorporates reference sequences in an attempt to differentiate the binding preferences of *N*-methylpyrroles to As and Ts [54]. Another proposal in characterizing genomic-binding sites of PI polyamides is the method known as cognate site identifier, or CSI [55]. CSI (Figure 4c) uses motif-finding algorithms to generate a positional map, from motif similarity scores, of all possible PIP-binding sites available in the sequence landscape defined by probe information on a microarray, then infers binding preference with a microarray data. CSI is arguably a variation of Bind-n-Seq and generates motif preferences, which one can indirect infer-binding information from reads gathered from a whole-genome sequencing experiment, with the caveat that the actual peak-calling process is still dependent on the choice of peak callers, and this motif information is merely used for annotation. While PIPs have experimentally demonstrated superior differentiating capabilities between full-match and mismatch motifs, an annotation-based approach such as Homer [56] is not sufficiently reliable for characterizing binding sites. With most PIPs being relatively short in base recognition, there may be upwards of a million hypothetical sites for typical design permutations, and one cannot fully exclude the possibility of coincidental motif overlapping, especially in polyamides designed to target oncogenes in cancer cell lines, in which the mutation profile of the specimen being sequenced can contain large variations from the reference genome. The presence of sequencing artifacts and the level of background noise may also reduce the accuracy of peak callers, subsequently affecting the final annotation results. Nevertheless, while Bind-n-Seq and its variations are highly capable in inferring the binding specificities of PIPs, their reliance on synthetic nucleotide libraries limits the scope of the coverage landscape; to overcome this issue, Bind-n-Seq needs to be complemented by chromatin or whole-genome sequencing methods that will allow a PIP’s actual in vivo binding locations to be thoroughly probed.

#### 3.2.2. PIP Co-Sequencing Methods: Generating a Lemma for Possible Genomic Effects

**Figure 5:**
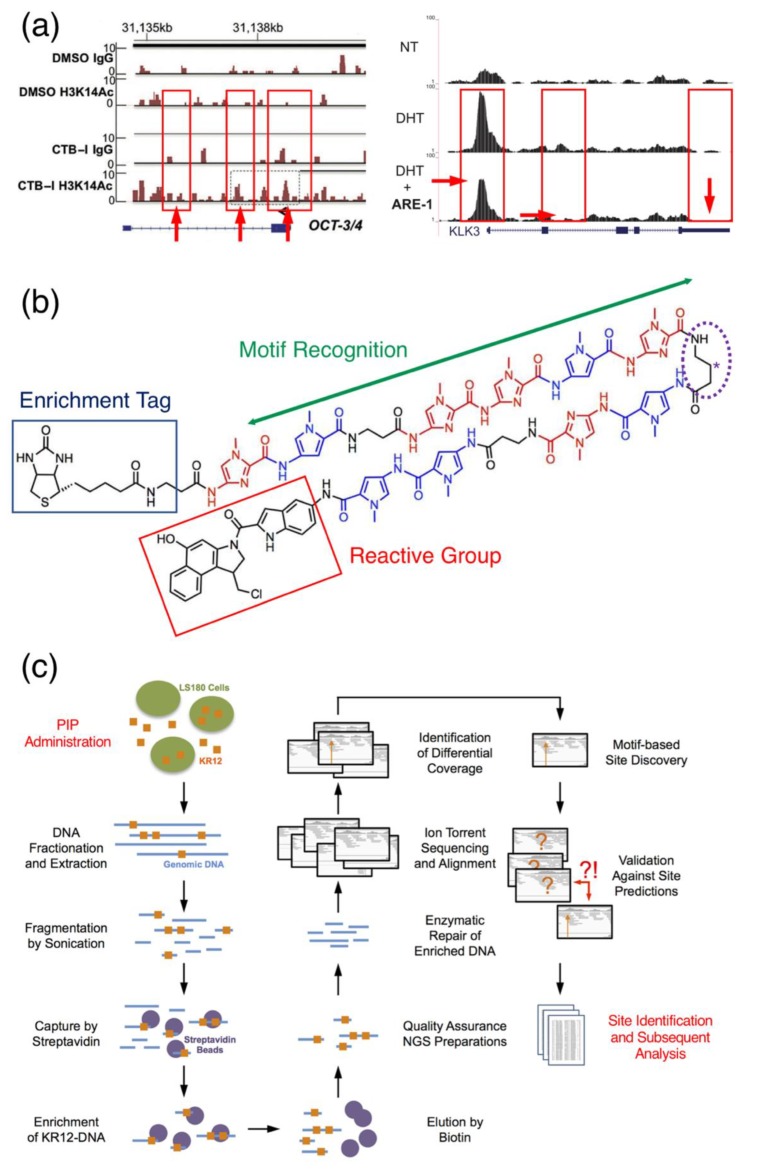
Sequencing with PIPs. (**a**) Examples of PIP-assisted ChIP-seq experiments using a CTB, a histone acetyltransferase activator, conjugated PIP in conjunction with ChIP-seq to detect changes in H3K14Ac activity in the promoter region of *OCT-3/*4 (left); another example of coupling dihydrotestosterone (DHT) with a PIP targeting the androgen receptor gene (*AR*) to evaluate changes in the genomic occupancy of *AR* in the promoter region of *KLK3* is provided on the right. *NT* indicates no treatment. Loss, gain or changes in amplitude of the local reads (red arrows and box with solid outlines) are then used to infer PIP genomic interference. Reproduced from [21], © 2015 Wiley (left) and [30], © 2019 Oxford University Press under the license terms of CC BY (right). (**b**) Scheme of a possible configuration of biotinylated PIPs for Chem-seq applications. In these situations, biotin is used as the enrichment tag (solid blue outline), and indole-*seco*-CBI (solid red outline) is the reactive alkylating moiety. The polyamide backbone provides a mean for the molecule to recognize and bind the minor groove of specific genomic DNA sites. Placement of the biotin enrichment tag at the hairpin turn (dotted purple outline labeled with asterisk) has also been proposed and tested [70]. (**c**) A representative Chem-seq workflow utilizing a bifunctionally conjugated PIP (see Figure 4b) to alkylate and enrich genomic-binding sites. Images for (**b**,**c**) were adapted from [65] by the author, © 2019 under the license terms of CC BY.

PIPs indeed have been used in conjunction with typical ChIP-seq applications to probe changes in the epigenetic landscape, such as the effect of a targeted histone acetyltransferase activator at the *OCT-3/4* loci (Figure 5a, left) [21] or the ability for a polyamide to perturb the promoter region of *KLK3* [30] (Figure 5a, right), although these methods do not directly indicate the location of PIP binding. Rather, they require local sequencing reads to be either manually inspected or require multiple control experiments to be performed so a differential gain or loss of features can be observed. In the case of Han et al., up to four tracks were necessary to elucidate the effect of a histone acetyltransferase activator-conjugated PIP [21]. These companion ChIP-seq experiments provide implied evidence of PIPs interacting with genetic regulatory elements near said sites, and the results, while useful, are unable to corroborate mechanistic hypotheses such as the displacement of histone upon minor-groove binding. In contrast, the usefulness of these co-sequencing experiments is that they dispense a comprehensive image of the genome through changes in certain epigenetic features, e.g., H3K27Ac, in the presence of a PIP. With the loss or gain of local peaks as a proxy for the influence of a PIP, these types of experiments deliver indirect and limited information on a polyamide’s exact genomic targets; to attain data on direct interacting targets necessitates the use of a PIP-centered sequencing method.

#### 3.2.3. Chem-seq: Discovery of In Vivo Genomic Binding Sites

Chem-seq is the catch-all terminology of mapping genome-wide interactions and target sites of small molecules. While the earliest adoption of such a term was perhaps Anders et al. [57], the terminology has also been extended to PIPs, with several publications exclusively employing “Chem-seq” to refer to the sequencing of DNA fragments captured by PIP. This method primarily involves the use of alkylating PIPs functionalized with moieties such as a biotin enrichment tag, typically at one of the polyamide backbone termini or the hairpin turn, along with an indole-*seco*-CBI-reactive group (Figure 5b), to provide anchoring via covalent linkage to the 3′ adenine of the target DNA. Should the polyamide be biotinylated, then precipitation with avidin resins can facilitate the isolation of these fragments for sequencing. Several reports have experimentally demonstrated the ability to enrich these fragments (Figure 5c), and we anticipate Chem-seq to be a versatile platform in the characterization of off-target effects for PIPs. A parallel variation of Chem-seq is COSMIC-seq [58], which is formulated upon the use of psoralen-conjugated PIPs for chromatin crosslinking and enrichment sequencing in conjunction to CSI. This method generates data that infer the location of cognate sites and is primarily focused on the monitoring and assessing of changes in the chromatin states; this method, however, is similar to the aforementioned combination of Bind-n-Seq with whole-genome sequencing in that it also indirectly maps the binding landscape with its motif-based approach.

At its current inception, aside from the need for further experimental optimization, Chem-seq faces a critical challenge with the subsequent data analysis. Those who encounter sequencing data have heard or made similar complaints: while data generation is straightforward, post-acquisition analysis is complicated. This is perhaps one of the reasons why few in the discipline spend significant effort on Chem-seq experiments: processing genomic data can be unforgiving, especially when the toolkits are insufficient. Next-generation sequencing data are noisy in nature; for instance, read duplication, natural or artificial, can potentially account for 30%-70% of the observed sequencing coverage [59]; additionally, the “blacklist” locations in the human genome typically found to produce anomalous and unreliable signal artifacts in sequencing experiments [60] span nearly 9% of the human reference genome (Appendix A), roughly three times the length of protein-coding genes [61]. Unlike proteins, PIP-DNA interactions are restricted to the minor groove; with their short motif recognition, the three-dimensional binding surfaces of PIP-DNA ligands will be smaller than typical protein-DNA ligands. Practically, peak callers written for ChIP-seq experiments will not be ideal in the analysis of Chem-seq data. This includes MACS [62], a popular model-based peak-calling tool ubiquitously used in ChIP-seq applications. Such a tool assumes that the distribution of peaks through the genome follows roughly a Poisson distribution process, and after shifting reads on the opposing strands, peaks are assumed to follow this distribution and be consequently called; with alkylating PIPs, the size of the binding site is essentially down to a single base, which exhibits wholly different binding interactions compared to typical transcription factors. This hypothesis, however, remains to be verified, since the single-base interaction surface located deep in the minor groove will render structural validation an exceedingly difficult task. As a matter of fact, even Anders’ original implementation had to utilize a lower enrichment threshold for Chem-seq, involving biotinylated AT7519, and the report was accompanied with generally lower magnitudes at the same sites [57], further highlighting the need for tailored computational tools in Chem-seq post-sequencing analyses.

The above hypothesis is hardly unique; various studies have compared the performance of a number of peak callers with different algorithmic backends in non-ChIP-seq experiments. Koohy and colleagues, for instance, found considerable discrepancies with DNase-seq data [63]; additionally, Poisson-based MACS has also been said to underperform in FAIRE-seq experiments compared to some of the other callers utilizing negative binomial models [64]. Certainly, while sequencing experiments such as ChIP-seq, DNase-seq and even Chem-seq generate the same type of short-read tags, differences in sequencer technology and the chemistry behind the enrichment of nucleotide fragments necessitate that the tools be at a minimum re-tune in order to achieve optimal performance. The differences in the level of background noise between different sequencing methods also cannot be ignored; DNase-seq is highly prone to noise and generates reads that are not strand-specific to have characteristic shifts that are often used to as a marker for positive peaks. Preliminary assessment of sequencing data suggested some similarities between Chem-seq and DNase-seq [65], but there have been no systematic studies to verify this to date.

Alternatives to a Poisson model-based approach include, certainly, the building of a different model. As with the case of ZINBA [64], negative binomial models may be suitable. However, considering the use of Bayesian models in computational biology, for instance, Spyrou and colleagues’ proposal [66] that Bayesian hidden Markov models can be used to detect genomic enrichments, increasing the model complexity that likely will improve the prediction outcome. A direct adaptation of the proposed Bayesian approach, which still depends on the presence of somewhat symmetric reads on both forward and reverse strands in situ (as a consequence of the larger protein-DNA-binding surfaces), will nonetheless require modifications, especially when most of the algorithms were developed with analyzing Illumina data in mind, not 100bp or longer, as they are today in the age of semiconductor (Ion Torrent) and long-read (PacBio) sequencers; this inevitably changes the peak distribution in Chem-seq data, such that extensive retooling is still required. With this kind of effort required, it is only imaginable that those in the discipline of PIP research will opt not to traverse down this path.

A tentative solution is to validate peak locations by the seemingly naïve assumption that true positive sites are enriched as the consequence of more affinity enrichment during the experiment, and thus, read enrichment is correlated. Under this assumption, one should be able to infer PIP-binding sites from their relative enrichment in the aligned reads. Based on this line of thinking, an approach is to utilize tools such as diffReps [67] or CRED [68], which, instead of a model-based approach, calculates read enrichments within differential sliding windows to deduce candidate sites. Existing studies utilizing MACS tended to underperform compared to enrichment-based methods at a high margin; for instance, we tested MACS on a CBI-conjugated PIP designed to bind G12D/V mutations of *KRAS* [65] and found that, on default settings, the model generated extremely unrealistic numbers of statistically significant candidates; furthermore, using a set of simulated Ion Torrent reads as the control, MACS also underreported nearly 200 sites compared to CRED [69]. This is not a criticism of MACS as a peak detection program but more of the point that, as complexity in sequencing data increases, no longer can we apply the same *lieu commun* assumptions in data analysis. With that said, enrichment-based approaches should nonetheless be coupled with secondary validations such as additional statistical validations over nonbinding sites of the same motif [65]. It is, however, important to note that, without moving away from motif-based approaches, the performance of enrichment-based peak calling will not fundamentally improve in Chem-seq applications, since the same issues continue to persist.

## 4. Perspectives

**Figure 6:**
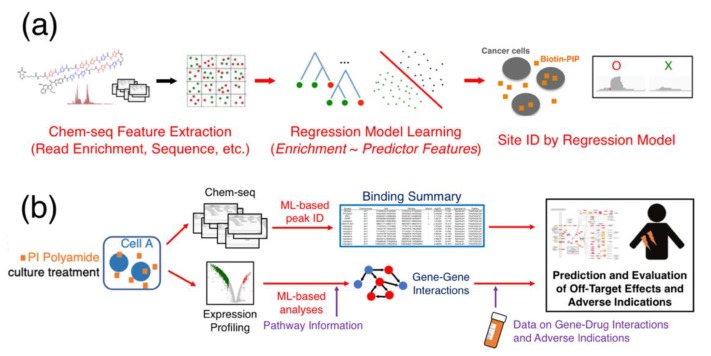
The use of machine learning to improve PIP off-target analysis from high-throughput data. (**a**) A proposal for using machine-learning methods to improve the site identification process in Chem-seq post-sequencing analysis. (**b**) A scheme of incorporating machine learning (ML) in characterizing genomic binding, as well as expression profiling data, to generate genetic interactions to elucidate the role of off-target effects for candidate PIPs.

What, then, will entice PIP researchers to head down this road not traveled? Certainly, the most direct method of reducing off-target effects with PIPs is to improve its binding affinity to its target, and approaches aimed at both increasing the recognition motif by conjoining multiple PIPs in tandem or modifying the base chemistry of the heterocycles will improve a polyamide’s affinity, but as its size approaches, the nuclear penetration limiting the law of diminishing returns is going to be well in effect. Alternatively, improvements in elucidating such details from expression profiling or Chem-seq data analysis by way of improving the computational analysis may perhaps be more viable. For instance, by leveraging the use of machine-learning methods to “study” Chem-seq peak characteristics (Figure 6a), the peak-calling process may see improved recognition. While Chem-seq peaks may exhibit nonuniform read distributions not fully expressible with the use of a single distribution model, there should nonetheless be key characteristics underlying the read pileup, and ensemble learning methods such as random forests [71] may be well-suited to recognize these feature characteristics. These methods “grow” a multitude of bifurcated decision trees that propagate through the various feature parameters during training, and consensus decisions from these ensemble of decision trees will generate reliable classification results based on the mode of the ensemble. Random forests are notably well-resistant to overfitting and, thus, can hypothetically create versatile predictor models across PIPs with small but generally similar major chemical structures and conjugated functional moieties.

Random forests see a large number of applications in computational biology, such as classification and regression problems of detecting evolutionary events [72], identifying DNA-binding proteins [73] and even predicting drug sensitivity [74]. In biology, where noise is a frequent issue, beyond the typical application of random forests in epidemiological studies for learning behavioral patterns among cohort statistics, this particular type of machine learning has also seen a spike in genomic studies [75]. Additionally, random forests generate deciding factors useful for estimating the relative importance of input parameters in a very natural way of explaining the observed phenomenon. These metrics, e.g., Gini importance scores [76], are often directly proportional to how frequent a particular feature is used to make decisions and can be translated in meaningful ways that directly illuminate the role of a particular biological feature. When coupled with enrichment-based callers, as mentioned in the previous section, machine-learning classifiers can be incorporated as a validation tool that is motif-independent.

We may also consider the use of deep learning, such as neural networks, to improve Chem-seq analysis. Neural networks of multiple layers of decision-making nodes are able to learn features in a dataset and have been used to infer gene expressions, and similar techniques have been applied to reduce noise in ChIP-seq data [77]. The ability to predict gene expressions [78], and epigenetic features such as chromatin structures [79], and enhancer sites [80] theoretically make deep learning also applicable in identifying peaks in Chem-seq data, considering the presence of high-background noise and nonuniform read distributions. Just as sequencing data can be thought of as images, they can be treated as inputs for convolutional neural networks and be processed similarly. For instance, if we recode sequence information as Boolean matrices of positions and features, we can then use layers of perception neurons to learn specific characteristics present in the Chem-seq data to predict the likelihood of a particular alignment being a positive peak.

An inherent problem with neural networks, however, is the cost of computing, especially as the number of neurons increases and the topology of the networks deepens. This problem is further exacerbated as model-training libraries increasingly offload the bulk of computations to graphical processing units (GPU), severely limiting code portability [81] and driving up development costs due to hardware preference. Another hurdle is the lack of well-constructed existing models that can be adapted to improve the learning results. “Transfer learning” is a method of deep learning that seeks to apply pretrained classifier models to a similar constructed problem. Recent advances in artificial intelligence have led to the explosion of image processing and learning models, such as ImageNet, Inception, VGG, etc. [82,83,84], all of which are extremely valuable in medical applications at identifying the edges of tumor and normal tissues in endoscopy, differentiating physiological features and so on [85]. These prebuilt models, however, may not be as useful for genomics data, which tend to have multiple output classifications, as well as large background noise and, subsequently, bias. A model erring on the side of caution in predicting a marginal peak as noise will tend to have lower false positives and artificially better performance, and in these situations, these models may not be useful at all.

As Frost epiphanies in *The Road Not Taken* [86], “two roads diverged in a wood, and I– I took the one less traveled by, and that has made all the difference,” at times, the decision of selecting a different path can change the trajectory of discovery. Minor-groove PIPs have made noticeable strides in the fields of cancer and disease biology, and their binding specificity and relative ease of administration have made them promising candidates in drug discovery; however, the mechanism behind their seeming ability for tumor homing and inhibitory actions remain poorly understood, and despite their specificity, very few have surfaced in clinical trials. Improvements made in the ability to analyze massive parallel-sequencing and expression-profiling data, for instance, by incorporating optimized machine-learning models to denoise Chem-seq data and predictor models for adverse indications (Figure 6b) can potentially aid researchers in understanding what the possible off-target effects may be for a new PIP. These efforts will certainly allow PIPs finally to be funneled into the process of lead development and improve their viability as drug candidates. At this point, unfortunately, few have elected for this road not taken; given the surprising progress of utilizing synthetic heterocycles as minor groove-binding PIPs today, there is something bound to be missed on the other path.

## Figures and Tables

**Table 1:** Common biophysical assays used to assess pyrrole-imidazole polyamide (PIP)-DNA binding in situations where one DNA sequence interacts to multiple PIPs or vice versa. “Response factor” describes the primary type of measurement collected for evaluating PIP-binding specificity in relation to off-target binding.

Assay	Purpose	Response Factor	Frequency of Use ^1^
Melting temperature	Comparison of PIP-dsDNA complex stabilities	ΔT_m_	+++
Gel-shift electrophoresis	Detection of whether a candidate PIP interacts with DNA containing a target-binding motif or otherwise	Changes in gel mobility	+++
Digital PCR	Comparison of the relative presence of PIP-DNA complexes by affinity enrichment, e.g., streptavidin-biotin interactions	Fold enrichment of PIP-DNA species	+
Surface plasmon resonance	Evaluation of adsorption and desorption rates to determine the kinetics of PIP-DNA and relative binding strength	Kinetic rate (e.g., *k_a_* or *k_d_*) and equilibrium constants (e.g., *K_D_*)	++
Circular dichroism	Monitoring of changes in DNA conformation structure upon PIP binding to discern differences in strength of the interaction with the minor groove	Changes in spectrometric signals within certain wavelengths	+

^1^ Relative frequency an assay is used to discern differences in PIP-DNA interactions; “+++” is the most common, followed by “++” and “+”.

## Appendix A

The claim that 9% of the human genome consists of “blacklisted regions” prone to producing artificially high signals is based on an estimate of the nonoverlapping length (approximately 274,970,000 bp) of all genomic segments annotated by Boyle et al. (version 2, released Nov. 2018) [87] compared to the length of the hg19 human genome (3,095,677,412 bp, excluding the mitochondrion, alternative haplotypes and random contigs).
